# Understanding entangled cerebral networks: a prerequisite for restoring brain function with brain-computer interfaces

**DOI:** 10.3389/fnsys.2014.00082

**Published:** 2014-05-06

**Authors:** Emmanuel Mandonnet, Hugues Duffau

**Affiliations:** ^1^Department of Neurosurgery, Hôpital LariboisièreParis, France; ^2^Department of Neurosurgery, Université Paris DiderotParis, France; ^3^IMNC, UMR 8165Orsay, France; ^4^Department of Neurosurgery, Gui de Chauliac Hospital, Montpellier University Medical CenterMontpellier, France; ^5^Team “Plasticity of Central Nervous System, Stem Cells and Glial Tumors,” INSERM U1051, Institute for Neuroscience of Montpellier, Montpellier University Medical CenterMontpellier, France

**Keywords:** brain networks, movement, language, anatomo-functional connectivity, functional restoration, brain-computer interface

## Abstract

Historically, cerebral processing has been conceptualized as a framework based on statically localized functions. However, a growing amount of evidence supports a hodotopical (delocalized) and flexible organization. A number of studies have reported absence of a permanent neurological deficit after massive surgical resections of eloquent brain tissue. These results highlight the tremendous plastic potential of the brain. Understanding anatomo-functional correlates underlying this cerebral reorganization is a prerequisite to restore brain functions through brain-computer interfaces (BCIs) in patients with cerebral diseases, or even to potentiate brain functions in healthy individuals. Here, we review current knowledge of neural networks that could be utilized in the BCIs that enable movements and language. To this end, intraoperative electrical stimulation in awake patients provides valuable information on the cerebral functional maps, their connectomics and plasticity. Overall, these studies indicate that the complex cerebral circuitry that underpins interactions between action, cognition and behavior should be throughly investigated before progress in BCI approaches can be achieved.

## Introduction

Technological advances in electrodes design have opened a number of new possibilities for brain-computer interfaces (BCIs) that decode large-scale neuronal activity (Lebedev and Nicolelis, 2006). It is now feasible to simultaneously record single-unit activity of hundreds of brain neurons, or record local field potentials (LFPs) from several relatively small brain areas. However, these technological achievements are insufficient for a BCI that restores a specific function, unless the *network* of brain areas involved in that function is well understood.

Although localizationist theories of brain function have been influential in the past, it is becoming increasingly clear that they are, out of date and of little use for BCIs (Nicolelis and Lebedev, 2009). Instead of assigning a fixed function to each discrete brain area, the current hodotopical and plastic view on cerebral organization states that brain functions are subserved by multiple cortical areas. These densely areas, interconnected by white matter pathways, work together rather than representing isolated processing units, and constitute a functional network.

Many fundamental issues of such functional networks are under debate. For example, is it possible to subdivide a cognitive task into separate elementary subtasks (performed successively or simultaneously), each associated with a spatially distinct subnetwork? Such spatial separability is supported by the observation of dissociation and double dissociations in patients with focal lesions (Shadmehr and Krakauer, 2008). Direct electrical stimulation (DES) is a powerful methodology for the investigation of spatial separability. For this purpose, DES is applied in awake subjects to induce transient deficits in particular components of cognitive tasks. Here, we review DES studies of motor and language functions and we discuss the relevance of these results to BCIs.

## Networks for voluntary movements

Since the seminal work of Penfield (Penfield and Bolchey, 1937), it has been well known that, under local anesthesia, cortical stimulation of the precentral gyrus evokes movements on the contra-lateral side of the body. This method reveals a cortical somatotopic map of the body, which is often called a “homunculus”. A similar homunculus can be reconstructed from cortical lesion studies. Based on these results, many neurological textbooks have adopted a simplified model of brain function and connectivity, which maps each brain area to a body part and assigns it a fixed function. For example, motor functions are assigned to an area located in the precentral gyrus and called the primary motor cortex (M1). M1 is considered as the lowest level of cortical motor hierarchy because all cortical motor signals converge. M1 then utilizes its somatotopic map to issue commands to spinal motoneurons, to which it is connected through the corticospinal tract, as well as less direct projections relayed by subcortical nuclei.

This model of M1 function is often mimicked by BCIs (Lebedev and Nicolelis, 2006). For example, Hochberg and his colleagues employed a 96-channel microelectrode array implanted in M1 of tetraplegic patients to interface cortical activity and a robotic arm (Hochberg et al., 2012). This study provided an important proof-of-concept demonstration, but the patients were unable to achieve good accuracy in the control of the robotic arm. This observation suggested that recording from M1 only may not be sufficient to capture all details of voluntary movements. One way to improve the performance of such neural prosthesis would be to implant multiple brain areas with recording arrays (BCIm for multiple brain computer interface), coding for distinct subparts of intentional movement, instead of just M1 (Lebedev and Nicolelis, 2006; Nicolelis and Lebedev, 2009). The performance would improve because neuronal signals provided by multiple areas better capture a diversity of neuronal mechanisms involved in programming and execution of voluntary movements. Hence, there is a growing understanding that BCIs may benefit from the recordings of large-scale motor networks and the utilization of such networks’ principles.

Important insights on the mechanisms of brain motor networks are provided by DES studies in humans. DES studies are conducted under three types of conditions: (i) pre-operative mapping with 50–60 Hz DES in pharmacoresistant epileptic patients; (ii) 60 Hz bipolar DES of gray and white matter during glioma surgery; and (iii) high-frequency deep brain stimulation (DBS) utilized for treatment of motor and psychiatric disorders. Motor responses can be evoked by DES of various cortical sites. Historically, Penfield and Bolchey (1937) reported that motor responses were not exclusively evoked from the precentral gyrus. For instance, they observed motor responses for about 25% of stimulated locations in the postcentral gyrus. Furthermore, they found that somatosensory responses were not localized to the postcentral gyrus either. They observed sensory responses in 25% of precentral recordings. Thus, these early experiments already questioned the segregation of sensory and motor functions in cortical areas traditionally believed to be purely motor or purely sensory. These results were somehow forgotten, but decisively rediscovered in 1996 (Nii et al., 1996). The functional significance of the mosaic pattern of sensory and motor representations within the primary motor and sensory areas remains to be elucidated.

Adding more sophistication to the function of somatosensory cortex, neurons in the primary motor and sensory areas exhibited responses to visual stimuli (Shokur et al., 2013). One influential theory explains such cross-modal responses in terms of a system of mirror neuron, first described in the premotor cortex of non-human primates and then in humans (Rizzolatti and Craighero, 2004). Mirror neurons respond to actions performed by a different person. Even in M1, neurons respond when a subject watches movement performed by somebody else (Vigneswaran et al., 2013). Despite these neurons having corticospinal projecting axons (pyramidal tract neurons), these M1 modulations do not evoke EMGs during observation only. This is possibly due to the fact that activation of M1 mirror neurons is accompanied by an inhibition of unwanted movement, for example unwanted imitation of the observed movements. Overall, these results indicate that M1 is not exclusively dedicated to motor execution. It has been suggested that M1 neurons that modulate their activity without producing overt movements can be utilized in BCIs (Schieber, 2011).

Apart from M1, motor responses can be evoked by DES applied to premotor areas. Premotor areas have direct connections to M1 *and* to the spinal cord (Dum and Strick, 1991). Premotor areas include four mesial (SMA proper, CMAr, CMAv, CMAd) and two lateral (PMd, PMv) areas (Dum and Strick, 2002). In humans, homologs of the mesial areas have been identified, and named SMA proper, RCZa, RCZp, and CCZ (Picard and Strick, 2001). DES of premotor areas induces movements, sometimes with complex pattern (Fried et al., 1991; Lim et al., 1996; Chassagnon et al., 2008; Basha et al., 2013).

The homologs of PMd and PMv are not clearly defined in humans, and stimulation of these areas often results in movement suppressions, called negative motor effects (Lüders et al., 1995; Mikuni et al., 2006). In negative motor areas (NMA), DES suppresses an ongoing movement on the contralateral side of the body. Such suppression can occur with or without associated speech arrest, and does not result in a loss of consciousness. As recently reviewed (Filevich et al., 2012), cortical NMA in humans have two epicenters: the pre-SMA (bilaterally) and the posterior part of the inferior frontal gyrus (right predominance). Additionally, several studies reported NMAs in PMd and PMv (Mikuni et al., 2006).

The physiological role of cortical NMAs is still debated. The classical interpretation is that 60 Hz stimulation may disturb neuronal networks responsible for sustained execution of movements. In this view, cortical NMAs would sustain the coding of (positive) motor programs that become jammed by DES. An alternative explanation is that these NMAs are physiologically involved in the inhibition of motor action; therefore their activation by DES inhibits movements. Note that pre-SMA and the IFG are not considered as premotor areas, meaning that this inhibition is probably not processed through direct projections to the spinal cord. We suggest that both interpretations are valid. In particular, the negative motor effect commonly elicited by the stimulation of the foot of the pre-central gyrus is likely due to a perturbation in the coding of the motor programs. This area is located just above the sylvian fissure, where Rizzolatti and Craighero reported the mirror neuron area of the ventral premotor cortex (vPMC; Rizzolatti and Craighero, 2004), as suggested in Mikuni et al. (2006). Because of the connections with parietal areas (Matsumoto et al., 2012, 2007), this site is an ideal candidate for the storage of *state estimation* (see the model of optimal feedback control, Shadmehr and Krakauer (2008)). Hence, 60 Hz signals can be viewed as an increase of the noise in this system, causing motor action to stop (by some yet undiscovered mechanism). On the other hand, there is also evidence that pre-SMA and IFG stimulation effect could be mediated by a direct activation of an inhibiting area (Filevich et al., 2012). In support of such dual mechanism, resection of vPMC induces definitive articulatory deficits if the underlying connectivity is not preserved (van Geemen et al., 2014), while no permanent deficits are observed following a resection of pre-SMA and right IFG.

We next ask how is DES-induced inhibition enacted: is there a decrease in the firing of M1 neurons or is there an increase in the firing in some inhibitory circuits? These details have been recently clarified using white matter stimulation in awake patients undergoing glioma resection. Stimulation within the depth of the posterior SMA and posterior part of middle frontal gyrus (dorsal premotor areas)—more or less at the level of the VAC line in a sagittal plane—stopped an on-going movement (Schucht et al., 2013). It was proposed that the effect was mediated by a direct activation of corticospinal neurons that inhibit motoneurons at the level of a spinal circuitry. This mechanism should not come as a surprise because cortical control over fine movements requires both supra-spinal excitatory and inhibitory modulation of the spinal motoneurons (Filevich et al., 2012). The cortical origin of these fibers fits well with the depth of the pre-central sulcus.

A second pathway, distinct from the previous one, has been shown to generate a specific disruption of bimanual movements, performed either in phase or in anti-phase (Rech et al., 2014). The spatial distribution of the corresponding DES sites is very close to the one cited above for unilateral negative motor responses. They lie close to the VAC line in the sagittal plane and could take their origin in the caudal cingulate zone or in the depth of the pre-central sulcus, and running towards the head of caudate nucleus and the anterior arm of internal capsule (Rech et al., 2014). Based on these studies, the concept of bimanual modulatory motor pathway was introduced, and it was suggested that the stimulated fibers could belong to the subcallosal fasciculus (Kinoshita et al., 2012), also called frontal aslant tract (Catani et al., 2012). But again, it remains currently speculative whether this bilateral inhibitory effect should be interpreted as noise that perturbs the normal functioning of the BMMP or whether DES triggers the physiological inhibitory mechanism of the BMMP.

In addition to DES studies, similar questions have been regarding the action of DBS, stimulation approach widely used to treat movement disorders, such as Parkinson’s disease. Here, computer modeling recently provided new insights. In these models, motor signs in parkinsonism are presumed to arise from the *bursting* activity of the GPi, which exerts an inhibitory effect onto the thalamus and compromises the fidelity of thalamocortical relay cells. This mechanism agrees with the recent suggestion that the thalamus is not simply a relay station but rather an active filter of the signals passing through cortico-thalamo-cortical loops, whose function is modulated by the basal ganglia output (Sherman and Guillery, 2011; Crosson, 2013). Computational simulations of the GPe-STN-GPi direct and indirect pathways showed that DBS can restore the thalamocortical transmission through *tonic* inhibition of the thalamocortical cells by GPi inputs, due to the prevention of the low-threshold calcium rebound bursts (Rubin and Terman, 2004; Guo et al., 2008; Rubin et al., 2012). Note that the pathological state is evoked by the *pattern* of activity within the network and not simply an average spiking neuronal rate in an area that correlates with the pathological state. Overall, computer simulation proved to be very helpful for better understanding of such networks.

In summary, we have a relatively good understanding of the motor system in non-human primates, but know very little about the organization and function of motor system in humans. Inferences from non-humans studies do not always hold true in humans. However, there is a growing understanding that motor systems in all primates function as distributed network of areas, each involved in multiple levels of motor control, rather than a collection of areas with specialized functions. Interestingly, DES studies in awake neurosurgical patients have provided important information on the organization of motor system in humans. Additional insights have been provided by computer modeling. We suggest that further research on the motor system in humans will be very beneficial for BCIs.

## Language networks

Unraveling the complex neural mechanisms of language is a real challenge. This challenge is even more difficult than the one for motor functions, because non-human primates cannot serve as a model. For many years, our knowledge came from the observations on speech deficits caused by brain lesions. These studies lead to the well-known model that describes a center of speech production (Broca’s area), a center of speech comprehension (Wernicke’s area), and a putative link between them made by the arcuate fasciculus. Following this model, the first BCI for language function utilized an array of microelectrodes implanted in the speech motor cortex (Kennedy et al., 2011).

Advances in non-invasive functional neuroimaging greatly enhanced our understanding of brain networks involved in language. It is now well recognized that distinct networks can be identified for different language aspects. In particular, a dual stream model of language has been proposed (Hickok and Poeppel, 2007). In this model, a left-lateralized dorsal stream is in charge of articulatory and phonological processes, whereas a bilateral ventral stream plays a central role in semantics. Observations of speech disturbances induced by axonal DES offer a unique tool to validate the model and to refine its axonal anatomical correlates. Indeed, errors induced by DES during a simple picture naming task, in awake patients operated for a glioma, allow to detect which subnetwork is transiently knocked out by the stimulation (Mandonnet et al., 2010). In these experiments, a speech therapist reports on-line the types of errors, e.g., dys- or anarthria, phonemic paraphasia, semantic paraphasia (Duffau et al., 2014). Dysarthria was observed when stimulating the opercular fronto-parietal loop that corresponds to the most lateral branch of superior longitudinal fasciculus (SLF III; Duffau et al., 2003). Phonemic paraphasia were elicited by stimulation of the arcuate branch of the SLF (Maldonado et al., 2011). Finally, semantic paraphasias were caused by stimulation of the inferior fronto-occipital fasiculus (IFOF; Duffau et al., 2005). On top of that, supramodal semantic abilities can also be tested by adding a semantic association test (PPTT) in the set of intraoperative tasks (Gatignol et al., 2004). Double dissociation patterns can be evidenced by alternating the picture naming test with the PPTT: during stimulation of deepest part of the IFOF, the patient can name the picture without being able to answer the PPTT, and conversely, during stimulation of a more superficial part of the IFOF, the patient has anomia while performing correctly the PPTT (Duffau et al., 2013; Moritz-Gasser et al., 2013). This disruption of semantic association can be viewed in the broader framework of an impairment of the noetic consciousness (Moritz-Gasser et al., 2013). Last but not least, the contextual rule linked to a given task is equally important. For example, the picture naming is not a *per-se* task: one could ask the patient to tell the color of the item, or to categorize items as animate/inanimate. The anatomical correlates of this contextual role and its interplay with the networks mentioned above still have to be discovered, although one can reasonably assume a major role of the deep gray nuclei (Gil Robles et al., 2005) and thalamus (Hebb and Ojemann, 2013).

Finally, studies in glioma patients provide important insights on the plasticity of language networks. Atlases of functional resectability provide a simple way to assign a quantitative value of plastic potential for each brain voxel (Mandonnet et al., 2007; Ius et al., 2011; De Witt Hamer et al., 2013) emphasizing the fundamental role of long-range connections for language processing, especially for the IFOF and SLF on the dominant side.

We suggest that future BCI applications for language function should take into consideration the new views on the neural mechanism of language function. In particular, it is important to consider that language processing is subserved by plastic, delocalized and synchronized networks that handle distinct language components. For example, in a locked-in patient with intact language networks, is it possible to decode words by interfacing a unique site or is it mandatory to use several recording devices, for example, in the phonological and semantic areas? At a first glance, it might seem advantageous to place the device in the speech motor cortex, which is the final encoding stage that generates speech output signal. However, the paradox is that this type of decoding may be way too complex. Indeed, since speech motor cortex encodes words at the level of muscles activity, this encoding includes information indirectly related to the targeted word (the speed, volume, timber at which the word is pronounced), which makes the decoding task very challenging. Hence, it might be much more efficient to interface areas that encode words at a higher level of abstraction, for instance its phonological (sequence of ordered phonemes) or semantic (either language modality dependant or even at the level of an amodal concept) representations. The problem when dealing with the higher level of coding is that the information is distributed over several interconnected areas, and one might not be able to retrieve the target word by recording a single area belonging to the network. One possibility would be to change the design of the electrodes so that we could record the area of convergence of the IFOF or arcuate fasciculus.

**Figure 1:**
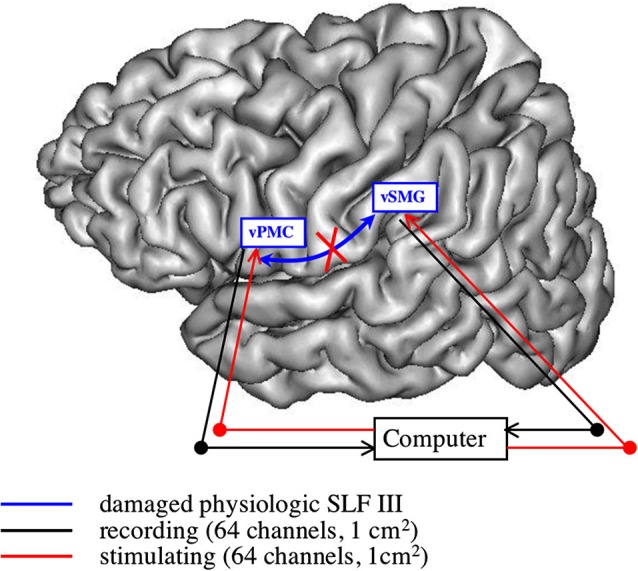
**Restoring the articulatory loop following white matter damage**. This subnetwork is constituted by the ventral premotor cortex (vPMC) (BA 6) and the (antero-)ventral supramarginal gyrus (vSMG), linked through the third branch of the superior longitudinal fasciculus (SLF III). When this pathway is damaged, with cortical areas still intact, one has to recreate a bidirectional synchronized link: stimulation pattern over one area is computed from the recorded activity in the other area, introducing a time lag (see for example protectJackson et al., 2006 for a short-range unidirectional version of such device).

Taking a different perspective, one can also envision BCI devices in aphasic patients following a stroke lesion, especially when one has to restore the long-range communication destroyed by the ischemia of the white matter. One possibility would be to build a brain-computer-brain interface (BCBI): two recording devices would be put in two distant disconnected areas still functional, and the activity of one area would shape the stimulation pattern over the other one, thus restoring a bidirectional synchronized link between both areas (see Figure 1).

## Conclusion

DES studies greatly contributed to unravel the complex issue of separability of motor and language functions. Of note, neural networks subserving mentalizing and emotion can also be mapped with this method (Herbet et al., 2014). Combining this methodology with neuronal recordings (cortico-cortical evoked potentials (Matsumoto et al., 2004, 2007, 2012; Swann et al., 2012; Enatsu et al., 2013)) is currently the best way to characterize anatomically and electrophysiologically each subnetwork underlying an elementary subfunction. However, it is anticipated that computer modeling will play an essential role in the analysis of experimental data. In turn, better knowledge of the electrophysiological activity within a subnetwork will pave the way towards new BCI concepts, including mBCI and BCBI. In summary, the entangled circuits underpinning interactions between action, cognition and behavior should need to be better understood for BCIs to take a full advantage of brain modulations in a reliable and reasonable way for patients.

## Conflict of interest statement

The authors declare that the research was conducted in the absence of any commercial or financial relationships that could be construed as a potential conflict of interest.

## Abbreviations

EMG: electromyogram
BCI: brain computer interface
SMA: supplementary motor area
CMAr: rostral cingulate motor area
CMAv: ventral cingulate motor area
CMAd: dorsal cingulate motor area
PMd: premotor dorsal
PMv: premotor ventral
RCZa: anterior rostral cingulate zone
RCZp: posterior rostral cingulate zone
CCZ: caudal cingulate zone
NMA: negative motor area
IFG: inferior frontal gyrus
VAC: vertical anterior commissure
BMMP: bimanual modulatory motor pathway
DES: direct electrical stimulation
PPTT: pyramid palm tree test
GPi: internal globus pallidum
GPe: external globus pallidum
STN: subthalamic nucleus
SLF: superior longitudinal fasciculus
IFOF: inferior fronto-occipital fasciculus
BCBI: brain-computer-brain interface
BCIm: multiple brain computer interface
DBS: deep brain stimulation
vSMG: ventral supramarginal gyrus.
